# A Simple Optical Aerosol Sensing Method of Sauter Mean Diameter for Particulate Matter Monitoring

**DOI:** 10.3390/bios12070436

**Published:** 2022-06-21

**Authors:** Liangbo Li, Ang Chen, Tian Deng, Jin Zeng, Feifan Xu, Shu Yan, Shu Wang, Wenqing Cheng, Ming Zhu, Wenbo Xu

**Affiliations:** 1 School of Electronic Information and Communications, Huazhong University of Science and Technology, Wuhan 430074, China; d202080751@hust.edu.cn (L.L.); chen_ang@hust.edu.cn (A.C.); tiandeng@hust.edu.cn (T.D.); zjcrystal@hust.edu.cn (J.Z.); m202072136@hust.edu.cn (F.X.); yanshu@hust.edu.cn (S.Y.); shuwang@hust.edu.cn (S.W.); chengwq@hust.edu.cn (W.C.); zhuming@hust.edu.cn (M.Z.); 2 Hubei Key Laboratory of Smart Internet Technology, Huazhong University of Science and Technology, Wuhan 430074, China

**Keywords:** particulate matter monitoring, Sauter mean diameter, mass concentration, light scattering

## Abstract

Mass concentration is a commonly used but insufficient metric to evaluate the particulate matter (PM) exposure hazard. Recent studies have declared that small particles have more serious impacts on human health than big particles given the same mass concentration. However, state-of-the-art PM sensors cannot provide explicit information of the particle size for further analysis. In this work, we adopt Sauter mean diameter (SMD) as a key metric to reflect the particle size besides the mass concentration. To measure SMD, an effective optical sensing method and a proof-of-concept prototype sensor are proposed by using dual wavelengths technology. In the proposed method, a non-linear conversion model is developed to improve the SMD measurement accuracy for aerosol samples of different particle size distributions and reflective indices based on multiple scattering channels. In the experiment of Di-Ethyl-Hexyl-Sebacate (DEHS) aerosols, the outputs of our prototype sensor demonstrated a good agreement with existing laboratory reference instruments with maximum SMD measurement error down to 7.04%. Furthermore, the simplicity, feasibility and low-cost features of this new method present great potential for distributed PM monitoring, to support sophisticated human exposure hazard assessment.

## 1. Introduction

The particulate matter (PM) monitoring technologies are playing important roles in indicating the exposure risk to adverse human health [1,2,3,4,5]. To quantify the risk, mass (volume) concentration is widely regarded as a key metric for PM monitoring [6,7,8,9]. However, recent toxicity studies indicated that, given the same mass concentration, particles of smaller size have a more destructive impact [4]. Smaller particles are more capable of penetrating into the alveolar epithelium and even translocating beyond the lung into the blood system than the bigger ones [5,10,11]. Additionally, the small particles, which have larger total surface area concentration than big particles at the same mass concentration, are easier to adsorb heavy metals and other toxins, which will cause more serious harm on biological tissues [12,13,14]. Therefore, it is insufficient to assessing PM by only using mass concentration, since the human exposure hazard of PM per unit mass concentration will increase significantly with the decrease of particle size.

The particle size distribution provides comprehensive information for evaluating the exposure hazard of PM per mass concentration. Existing particle size distribution (PSD) analysis technologies have been widely used in laboratories for aerosol scientific research, such as aerodynamic, electrical mobility, Fraunhofer diffraction [15,16,17], and particle count [18], etc. However, these laboratory-grade instruments are prohibitively expensive and bulky for large-scale consumer applications.

Comparing with above-mentioned instruments, low-cost and distributable sensors are more desirable for PM monitoring and exposure hazard studies. Most of the low-cost sensors are based on optical methods. The equivalent concentration and particle size obtained with lost-cost sensors may deviate from the metric of interest, such as mass concentration and aerodynamic diameter. American Eco-Chem [19] developed a portable instrument based on electric charge measurement technology to obtain the surface area concentration of aerosols. Robert T et al. measured the surface area concentration and lung deposition equivalent size of PM between 44 nm and 1050 nm based on electrical mobility technology to monitor ultra-fine particle characteristics [11]. Kwon et al. developed a Microfluidic Nanoparticle Analysis Chip [20] to measure number concentration of nanoparticles on the basis of electrostatic classification methods. On top of that, optical methods can further compact the volume and extent the maintenance intervals due to the simple structure, highly integrated optical components and non-contact measurement. Greenberg et al. designed a Multi-Parameter Aerosol Scattering Sensor (MPASS) [21,22] to measure the surface area concentration and volume concentration with limited range from 100–1000 nm based on a single-wavelength incident laser source and dual observing angles. However, there haven’t been any effective optical sensing methods to provide information about particle size. Thus, an aerosol sensing method with high accuracy and wide measurement range for PM monitoring is on demand.

The SMD is defined as the statistical mean size of particles that have the same volume/surface area ratio [23,24]. It provides a unique ability to describe the particle aerodynamic deposition distribution in the respiratory system and alveolar penetration rate related to the particle size, as well as the toxins adsorption related to the surface area concentration. Consequently, SMD is an applicable metric for the exposure hazard assessment of PM per unit mass concentration. In this work, a simple SMD sensing method is proposed for PM monitoring based on 4-channel light scattering signals. The SMD and mass concentration are calculated via a non-linear model, which eliminates the effect of particle size distributions and refractive indices. By using customized dual-wavelength LEDs, a simple-structured prototype sensor is fabricated for performance evaluation. According to the experimental results of DEHS aerosols of different sizes, the maximum measurement error of SMD is down to 7.04% while that of mass concentration is 23.68%. With the extra characterization ability of the particle size and surface area via SMD, this method can be implemented for comprehensive hazard assessment of PM at a sufficiently low cost in widespread deployment applications.

The contributions of this study are as follows:

(1) Firstly, SMD was proposed for the hazard assessment of PM per unit mass concentration, which comprehensively reflects the aerodynamic deposition distribution, alveolar penetration rate and toxins adsorption in the aspects of both particle size and surface area concentration.
(2) A simple optical sensing method was developed to measure the SMD. A non-linear conversion model is established to precisely calculate the SMD of aerosols with different PSD, while the measurement accuracy of the volume concentration is also improved significantly.
(3) A low-cost and portable proof-of-concept sensor was designed and fabricated by using multiple scattering signals with optimized optical parameters. The simulation and experimental results show that our sensor can precisely measure the SMD and the volume concentration of the aerosol samples.
(4) The sensor is applicable for various PM hazard assessment researches, source attribution investigations, epidemiological studies, and other applications.

The rest of this paper is organized into four sections: Section 2 formulates the problem and presents the sensing method of SMD with a non-linear model using multiple scattering signals. Section 3 shows experimental results of the prototype sensor with mono-disperse DEHS aerosol samples, and simulation results of organic matter (OM), black carbon (BC), and dust. Section 4 concludes the whole paper.

## 2. Materials and Methods

### 2.1. The Definition of SMD of Particulate Matter

Smaller particles, which have stronger suspension ability and larger surface area concentration, can deposit deeper in human respiration system and carry more toxins into the body. In order to indicate the difference of surface area concentration and particle size, SMD is defined as [25,26,27,28]:

(1) $$\mathrm{SMD}=6\frac{C_{V}}{C_{S}}=\frac{6}{\rho }\frac{C_{M}}{C_{S}}$$

where $C_{V}$ is the volume concentration of PM, $C_{S}$ is the surface area concentration, $C_{M}$ is the mass concentration, ρ is a constant value representing the density of PM [29]. As described in Equation (1), SMD indicates the particle size information of PM. Once the mass concentration is given, an explicit mathematical relation between SMD and $C_{S}$ can be established. Therefore, both the particle size and surface area concentration information can be obtained simultaneously. For instance, a small SMD implies that the PM mainly composes of small particles, and has a large surface area concentration relatively. Hence, with particle size and surface area information, the SMD can be easily obtained as a metric for the hazard assessment of PM per unit mass concentration.

### 2.2. The Sensing Method of SMD Based on Light Scattering

To achieve non-destructive measurement, fast response, and low cost, we conducted the detection of SMD by using scattered light as the received signal. As demonstrated by Hulst et al. in [30], the light intensity *P* scattered by the PM sample can be described as:

(2) $$P=\int C_{N}f\left(x,\mu ,\sigma \right)q\left(x,m,\lambda ,\theta \right)dx$$

where $C_{N}$ is the number concentration of PM, q(x,m,λ,θ) describes the intensity of monochromatic light scattered by a single particle, λ and θ denote the wavelength of incident light and the observing angle respectively. *m* is the refractive index of aerosols, and f(x,μ,σ) is the PSD of aerosol samples, which can be described by the logarithmic-normal distribution model [27]:

(3) $$f\left(x,\mu ,\sigma \right)=\frac{1}{\sqrt{2\pi }x\ln\sigma }\exp\left[-\frac{{\left(\ln x-\ln\left(\mu \right)\right)}^{2}}{2\ln^{2}\sigma }\right]$$

where *x* is the equivalent spherical diameter within the particle size distribution range of PM, μ denotes the count median diameter (CMD), and σ describes the geometric standard deviation (GSD). According to the work of Kulkarni et al. in [31], the light intensity *q* of a single particle changes with the relative relationship between the particle size *x* and the wavelength λ.

**Figure 1:**
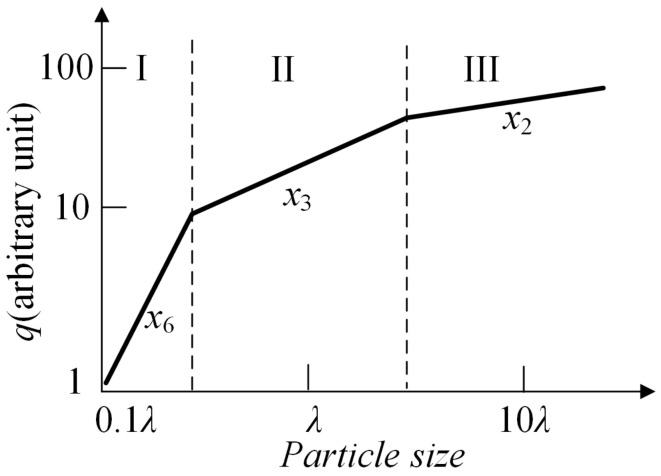
The general relationship between scattering intensity and particle size.

As illustrated in Figure 1, the light intensity *q* can be respectively described as:

(4) $$q\left(x,m,\lambda ,\theta \right)\approx \left\{\begin{array}{cc} T_{I}\cdot x^{6}, & \mathrm{when}\;x<\lambda \\ T_{II}\cdot x^{3}, & \mathrm{when}\;x\approx \lambda \\ T_{III}\cdot x^{2}, & \mathrm{when}\;x>\lambda \end{array}\right.$$

where $T_{I}$, $T_{II}$, and $T_{III}$ are the transformation factors between *q* and *x*. $T_{I}$, $T_{II}$, and $T_{III}$ are also related to the refractive index *m*, the observing angle θ, λ and *x*. Thus, the scattered light signal $P_{{\left[\lambda ,\theta \right]}_{S}}$ for a short wavelength can be described as:

(5) $$P_{{\left[\lambda ,\theta \right]}_{S}}=\int C_{N}f\left(x,\mu ,\sigma \right)T_{III}x^{2}dx=C_{N}T_{III}\int f\left(x,\mu ,\sigma \right)x^{2}dx$$

Similarly, for a long wavelength, the scattered light signal $P_{{\left[\lambda ,\theta \right]}_{L}}$ can be approximatively described as:

(6) $$P_{{\left[\lambda ,\theta \right]}_{L}}=\int C_{N}f\left(x,\mu ,\sigma \right)T_{II}x^{3}dx=C_{N}T_{II}\int f\left(x,\mu ,\sigma \right)x^{3}dx$$

Thus, the SMD described by Equation (1) can be calculated by:

(7) $$\begin{array}{cc} \mathrm{SMD} & =6\frac{C_{V}}{C_{S}}=6\cdot \frac{\int \frac{4}{3}\pi {\left(\frac{x}{2}\right)}^{3}f\left(x,\mu ,\sigma \right)dx}{\int 4\pi {\left(\frac{x}{2}\right)}^{2}f\left(x,\mu ,\sigma \right)dx}=\frac{\int f\left(x,\mu ,\sigma \right)x^{3}dx}{\int f\left(x,\mu ,\sigma \right)x^{2}dx}\\ \; & =\frac{P_{{\left[\lambda ,\theta \right]}_{L}}/\left(C_{N}T_{II}\right)}{P_{{\left[\lambda ,\theta \right]}_{S}}/\left(C_{N}T_{III}\right)}=\frac{T_{III}}{T_{II}}\cdot \frac{P_{{\left[\lambda ,\theta \right]}_{L}}}{P_{{\left[\lambda ,\theta \right]}_{S}}}=T_{\mathrm{SMD}}\frac{P_{{\left[\lambda ,\theta \right]}_{L}}}{P_{{\left[\lambda ,\theta \right]}_{S}}} \end{array}$$

where $T_{\mathrm{SMD}}$ is the transformation factor between the SMD and the ratio of scattered light signals, *x* is the equivalent spherical diameter. However, $T_{\mathrm{SMD}}$ will irregularly fluctuate with the change of the PM parameters ${\left[m,f\left(x,\mu ,\theta \right)\right]}_{i}$ and the optical parameters ${\left[\lambda ,\theta \right]}_{j}$. To eliminate the impact, a modified model of $T_{\mathrm{SMD}}$ is established to dynamically adjust $T_{\mathrm{SMD}}$ for each aerosol sample.

**Figure 2:**
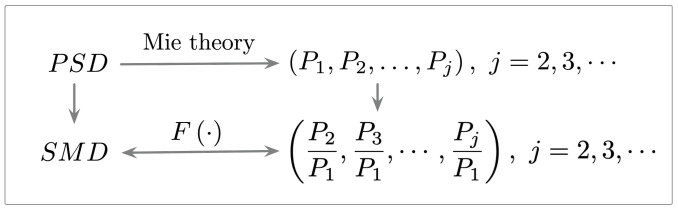
The derivation scheme of $T_{\mathrm{SMD}}$.

As depicted in Figure 2, according to the Mie theory, we can calculate the intensity ($P_{1},\ldots ,P_{j}$) of the scattered light for each channel on the basis of the PSD information and refractive indices. Therefore, the relation between $T_{\mathrm{SMD}}$ and scattered light intensity ($P_{1},\ldots ,P_{j}$) can be established as follows:

(8) $$T_{\mathrm{SMD}}=F\left(\frac{P_{2}}{P_{1}},\frac{P_{3}}{P_{1}},\cdots ,\frac{P_{j}}{P_{1}}\right),j=2,3,\cdots$$

where, $P_{j}$ is the scattering light signal with different wavelength $\lambda (\lambda \in \left[\lambda_{S},\lambda_{L}\right])$ or observing angle θ. As illustrated by Equation (8), a vector space is built to map the $T_{\mathrm{SMD}}$s of all potential PM samples by the ratios of optical channels based on the modified model, where the influence of number concentration $C_{N}$ is eliminated by the optical channel ratio. In this vector space, if the potential PM sample is uniquely identified by the ratios of optical channels, $T_{\mathrm{SMD}}$ is determined and the corresponding SMD can be measured accurately. Meanwhile, if there are several potential PM samples with similar ratios of optical channels, then the $T_{\mathrm{SMD}}$ cannot be identified and should be substituted by the average value as a compromise, and the corresponding SMDs will have a certain measurement deviation. In consideration of the sampling error of the optical signals, we have added 5% random noise to the optical signal of each channel.

As mentioned above, more optical channels will be useful in improving the measurement performance of our method. However, it will also increase the complexity of the measuring system. To determine appropriate optical parameters ${\left[\lambda ,\theta \right]}_{j}$, the SMD measurement relative standard deviation (RSD) is defined to indicate the SMD measurement accuracy of different optical parameters:

(9) $$RSD=\sqrt{\frac{1}{i}\sum \frac{{\left({\mathrm{SMD}}_{real}-{\mathrm{SMD}}_{test}\right)}^{2}}{{\mathrm{SMD}}_{real}^{2}}}$$

where *i* is the number of PM samples. ${\mathrm{SMD}}_{real}$ is the real SMD, ${\mathrm{SMD}}_{test}$ is the SMD calculated according to Equations (7) and (8). Based on Equation (9), a constraint model is built to optimize optical parameters by minimizing the RSD as:

(10) $$\left[\lambda_{L},\lambda_{S},\theta ,j\right]=\arg\min\mathrm{RSD},\mathrm{st}.\;\mathrm{in}\;j\;\mathrm{channels}$$

### 2.3. The Optimization of Optical Parameters

Considering the availability of light sources, 950 nm infrared LED is selected as the light source of long wavelength $\lambda_{L}$, while 450 nm blue LED is selected as the light source of short wavelength $\lambda_{S}$. Once the wavelength is determined, the measurement accuracy of SMDs is only affected by the observing angle θ and the number of optical channel *j*. Thus, a simulation experiment is conducted to optimize the observing angle θ with the different number of channels *j* according to Equation (10). As shown in Table 1, the maximum channel number *j* is limited to 5 in consideration of the structural complexity. Meanwhile, due to the limitation of mechanical design and the interference caused by stray light, the optional observing angle is restricted from forward 40° to backward 140° with a stride of 5°.

On the other hand, three kinds of typical PM (organic matter (OM), black carbon (BC), and dust) are taken into consideration for the optical parameters optimization. The corresponding refractive indices are 1.53+0.001i, 1.95+0.79i, and 1.53+0.001i respectively [29]. Considering the settlement of large particles of size >10,000 nm, the particle size distribution range *x* is set from 10 to 10,000 nm with an interval of 10 nm. The CMD μ is set to be [100,2500] with an interval of 25 nm for human main activity area [10]. Meanwhile, the GSD σ typically ranges from 1.5 to 2.0 with an interval of 0.1 [27]. In addition, to present a laboratory verification of our method, the mono-disperse DEHS aerosol test samples are also taken into consideration during the optimization of optical parameters. The particle size distribution range and the CMD of DEHS are consistent with the PM listed in Table 2, while the GSD is set from 1.1 to 1.4 with the interval of 0.1, and the refractive index is 1.45. The PM and DEHS parameters mentioned above are all listed in Table 2.

According to the optional optical parameters in Table 1, the optical parameter sets with the different number of measurement channels are optimized by minimizing the RSD according to Equation (10). As shown in Table 3, the minimum RSD decreases obviously with the number of optical channels increasing from 2 to 3, and slowly when the channel number continues to increase from 3 to 5. Since the minimum RSD decreases very slightly from 4 to 5 channels, and the complexity of 5 channels is much higher than that of 4 channels, a 4-channel detection structure with ${\left[450\;\mathrm{nm},40^{\circ }\right]}_{1},{\left[950\;\mathrm{nm},40^{\circ }\right]}_{2},{\left[450\;\mathrm{nm},115^{\circ }\right]}_{3},{\left[950\;\mathrm{nm},125^{\circ }\right]}_{4}$ is adopted to measure the SMDs of PMs, where the corresponding RSD is 6%.

### 2.4. The Design of Prototype Sensor

To verify the effectiveness of our method, a prototype sensor is designed and built for experimental test. As mentioned in Section 2.3, $\left[450\;\mathrm{nm},40^{\circ }\right],\left[950\;\mathrm{nm},40^{\circ }\right],\left[450\;\mathrm{nm},115^{\circ }\right]$, $\left[950\;\mathrm{nm},125^{\circ }\right]$ are selected as the optimal optical parameters. To further simplify the structure of the sensor, an integrated dual-wavelength LED of 450 nm and 950 nm is adopted as the light source instead of single-wavelength LED. Hence, we re-optimized the corresponding observing angles to minimize the SMD measurement error according to Equation (10).

**Figure 3:**
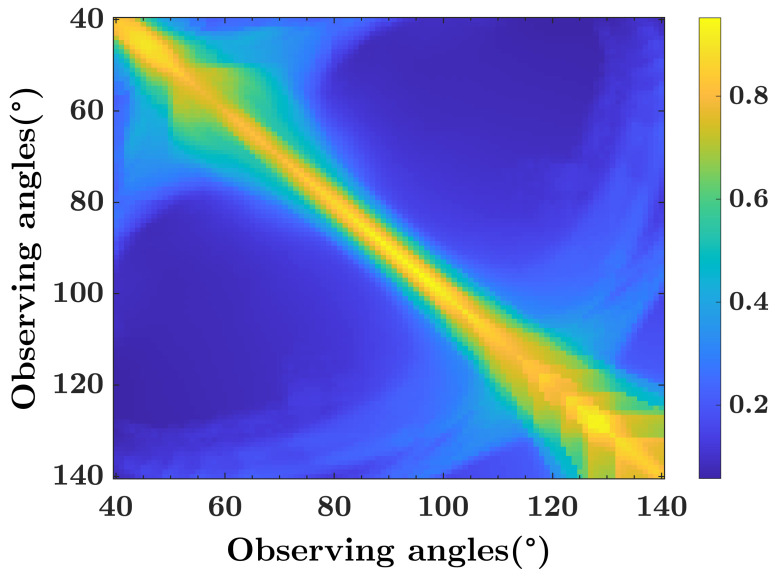
Relative standard deviation versus observing angles with dual-wavelength LED.

**Figure 4:**
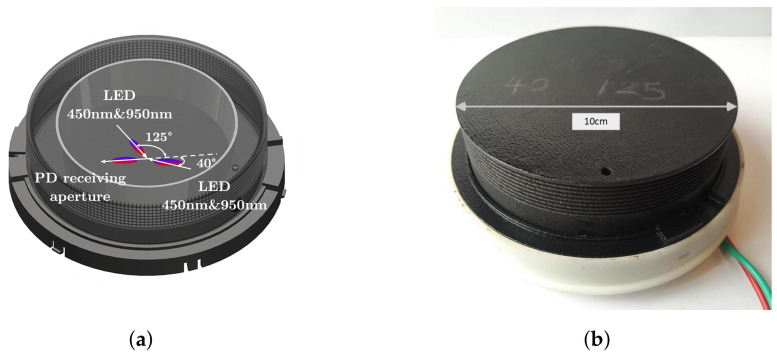
The design of the prototype sensor. (**a**) The 3D model of sensor. (**b**) The prototype sensor.

As shown in Figure 3, the RSDs are indicated by the color bar, where the horizontal axis and vertical axis indicate the two observing angles of the dual-wavelength LEDs. According to the simulation results, the observing angles 40° and 125° are selected, where the corresponding minimum RSD is 6%. Comparing with single-wavelength diode, the RSD of the re-optimized channels just increases slightly while the optical structure is significantly simplified. Thus, a 4-channel prototype sensor [450 nm & 950 nm @40${}^{\circ }$], [450 nm & 950 nm @125${}^{\circ }$] is designed, as shown in Figure 4a. With the simple and low-cost design, the prototype sensor is manufactured by 3D printing with size of diameter 10 cm × height 6 cm, as shown in Figure 4b.

### 2.5. The Establishment of the Experimental Platform

**Figure 5:**
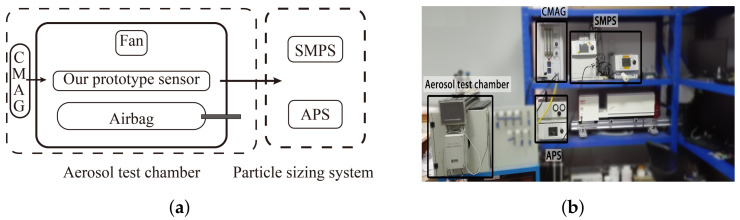
The experimental platform. (**a**) Block diagram of aerosol chamber and particle sizing system. (**b**) Photograph of aerosol samples testing system and aerosol sizing system.

To evaluate the performance of our prototype sensor, an experimental platform is established. As shown in Figure 5, the platform is composed of two parts: Aerosol testing system and aerosol sizing system. In aerosol testing system, the prototype sensor is placed in the aerosol chamber, where the aerosol samples are well stirred by the muffin fans. An air bag is embedded in the chamber to keep particle concentration and air pressure stable. Our prototype sensor was tested with DEHS samples of different SMDs generated by the CMAG-3475 (Condensation Mono-disperse Aerosol Generator, TSI). In aerosol sizing system, SMPS-3936 (Scanning Mobility Particle Sizer Spectrometer 3936, TSI) and APS-3321 (Aerodynamic Particle Sizer Spectrometer 3321, TSI) are used as the reference instruments to measure the concentrations and the SMDs of the aerosol samples.

## 3. Results and Discussion

Based on the experimental platform, the 4-channels prototype sensor of [450 nm & 950 nm, 40°]${}_{1-2}$, [450 nm & 950 nm, 125°]${}_{3-4}$ is tested by mono-dispersed DEHS aerosols. Severn types of DEHS aerosol samples of different SMDs were measured by our prototype sensor and the reference instruments.

**Figure 6:**
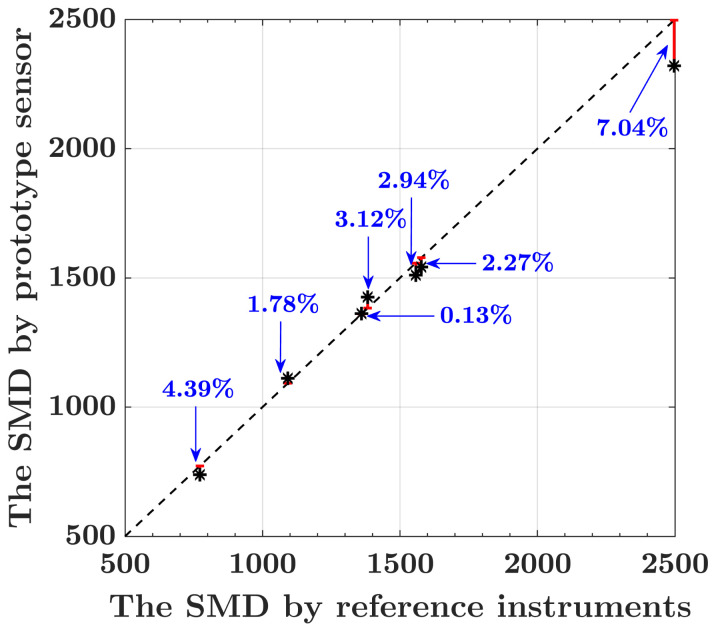
The mass concentration of DEHS samples obtained by our prototype sensor versus the reference instruments.

As shown in Figure 6, the experimental results indicate that the maximum measurement error of the SMD is 7.04%, where the horizontal axis is the SMD measured by the reference instruments, and the vertical axis is the SMD obtained by our sensor.

On the other hand, similar to the existing single-channel photoelectric PM sensor, our method can also measure the mass concentration according to the intensity of the scattering light signal. Furthermore, with the help of the particle size distribution information characterized by the SMD, the mass concentration measurement is correctly measured with dynamically corrected mass concentration conversion coefficient. Compared with the single-channel sensing method, the measurement accuracy of the mass concentration is also significantly improved. Table 4 shows the mass concentration measured by our prototype sensor, the reference instruments and existing single-channel sensor. Comparing with the mass concentration measured by a single channel, the errors are significantly reduced, where the maximum measurement error of the mass concentration is reduced from 66.67% of single channel sensor to 23.68% of our prototype sensor. The measurement error of mass concentration can be further reduced if mass concentration is taken into consideration during optical structure optimization at the expense of increasing SMD measurement error. A balance algorithm between these two parameters is an important topic for further study.

**Figure 7:**
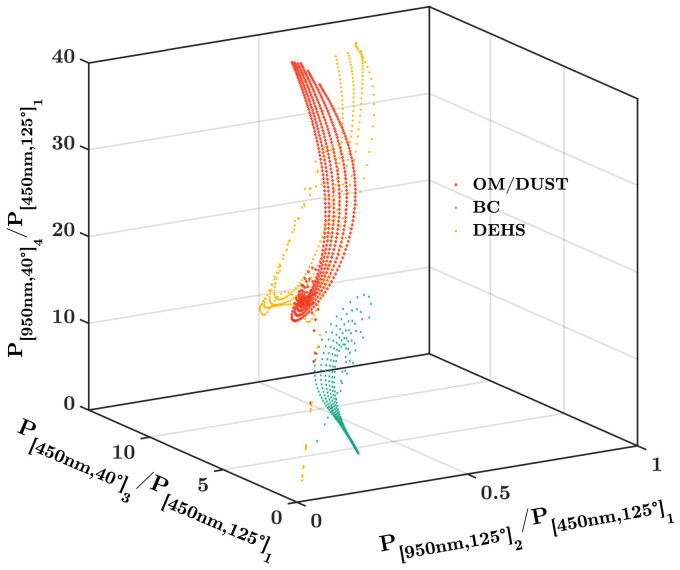
The $T_{\mathrm{SMD}}$ of aerosol samples with different particle size distributions are mapped in the ratio vector space of scattering signals.

In order to further evaluate the performance of our method for more SMDs, all PM samples listed in Table 2 and DEHS aerosols were tested by the simulation experiments. As shown in Figure 7, the $T_{\mathrm{SMD}}$s are mapped in the vector space formed by the 3 ratios, which are calculated from the 4 optical channels. The dots in colors indicate different types aerosol samples. Thus, the SMD can be obtained according to Equation (7).

**Figure 8:**
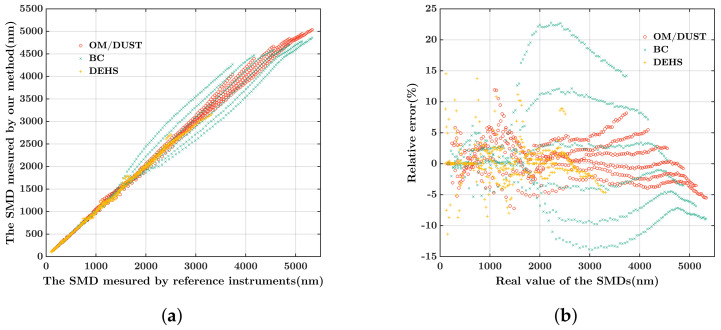
The performance of our prototype sensor evaluated by the simulation experiments. (**a**) The contrast between ${\mathrm{SMD}}_{real}$ and ${\mathrm{SMD}}_{test}$. (**b**) The relative measurement error of aerosol samples.

According to Figure 7, we tested the performance of our prototype sensor to measure aerosol samples of different PSDs. As shown in Figure 8a, the horizontal axis represents the ground truth of SMDs, and the vertical axis represents the SMD value retrieved by our method. Compared to BC samples, our method shows better performance for OM, DUST, and DEHS aerosols. According to Equation (9), the combined RSD of all PM samples and DEHS aerosols is 6%. Figure 8b shows the measurement accuracy of each sample, where the horizontal axis is the SMDs of samples, the vertical axis is the relative measurement errors. As shown in Figure 8b, the relative error ranges from −14% to 23% for all aerosol samples including OM, dust, BC and DEHS.

As discussed above, the experimental and simulation results prove that our method can measure the SMD and mass concentration of aerosol samples accurately at the same time. Thus, our method can provide another key metric to support more comprehensive PM exposure hazard assessment.

## 4. Conclusions

In this paper, a method based on light-scattering is proposed to measure the SMD of PM besides the mass concentration. A prototype sensor with 4 channels is built to measure the SMD of PM with optimized optical parameters [450 nm & 950 nm, 40°]${}_{1-2}$, [450 nm & 950 nm, 125°]${}_{3-4}$. The RSD of SMDs reaches as low as 6% in the simulation experiments, while the maximum relative measurement error is 7.04% in DEHS tests. Meanwhile, the experimental results show that our sensor can also measure the mass concentration of DEHS aerosol accurately. Therefore, with a simple design and the extra ability of detecting particle size and surface area characterizing based on the SMD, our sensing method demonstrates great potential to be implemented for various PM hazard assessment researches, source attribution investigations, epidemiological studies, and other applications.

## Figures and Tables

**Table 1:** The optional optical parameters.

Channel Number *j*	Incident Light Wave Lengths λ (nm)	Observing Angle θ (°)
2:1:5	$\left\{\lambda_{S}=450,\lambda_{L}=950\right\}$	4:5:140

**Table 2:** The PM and DEHS parameters in human main activity areas.

Aerosol Type	Particle Size Distribution Range (nm)	Count Median Diameter μ (nm)	Geometric Standard Deviation σ	Refractive Index *m*
OM & Dust	10:10:10,000	100:25:2500	1.5:0.1:2.0	1.53+0.001i
BC	10:10:10,000	100:25:2500	1.5:0.1:2.0	1.95+0.79i
DEHS & Dust	10:10:10,000	100:25:2500	1.1:0.1:1.4	1.45

**Table 3:** The optimal optical parameters.

Channel Number *j*	2	3	4	5
${\left[\lambda ,\theta \right]}_{j}$	${\left[450\;\mathrm{nm},70^{\circ }\right]}_{1}$ ${\left[950\;\mathrm{nm},80^{\circ }\right]}_{2}$	${\left[450\;\mathrm{nm},70^{\circ }\right]}_{1}$ ${\left[950\;\mathrm{nm},40^{\circ }\right]}_{2}$ ${\left[450\;\mathrm{nm},115^{\circ }\right]}_{3}$	${\left[450\;\mathrm{nm},40^{\circ }\right]}_{1}$ ${\left[950\;\mathrm{nm},40^{\circ }\right]}_{2}$ ${\left[450\;\mathrm{nm},115^{\circ }\right]}_{3}$ ${\left[950\;\mathrm{nm},125^{\circ }\right]}_{4}$	${\left[450\;\mathrm{nm},40^{\circ }\right]}_{1}$ ${\left[950\;\mathrm{nm},40^{\circ }\right]}_{2}$ ${\left[950\;\mathrm{nm},115^{\circ }\right]}_{3}$ ${\left[450\;\mathrm{nm},135^{\circ }\right]}_{4}$ ${\left[950\;\mathrm{nm},140^{\circ }\right]}_{5}$
RSD	70%	9%	6%	6%

**Table 4:** Data of the mass concentration of DEHS samples obtained by our prototype sensor versus the reference instruments.

Groups	1	2	3	4	5	6	7
Reference	97.20	90.70	130.00	108.00	149.00	107.00	175.00
Single Channel & Relative Error	162 (66.67%)	126 (38.92%)	115 (11.54%)	105 (2.78%)	87 (41.61%)	78 (27.10%)	93 (46.84%)
Prototype Sensor & Relative Error	81.04 (16.63%)	96.09 (5.94%)	123.35 (5.12%)	123.54 (14.39%)	113.71 (23.68%)	110.91 (3.65%)	212.53 (21.45%)

## Data Availability

Not applicable.
